# Improving SDG Classification Precision Using Combinatorial Fusion

**DOI:** 10.3390/s22031067

**Published:** 2022-01-29

**Authors:** D. Frank Hsu, Marcelo T. LaFleur, Ilyas Orazbek

**Affiliations:** 1 Laboratory of Informatics and Data Mining, Department of Computer and Information Science, Fordham University, New York, NY 10023, USA; iorazbek@fordham.edu; 2 Department of Economic and Social Affairs, United Nations, New York, NY 10017, USA

**Keywords:** cognitive diversity, combinatorial fusion algorithm (CFA), LDA, rank combination, rank-score characteristic (RSC) function, score combination, semantic web, sustainable development goals (SDGs), topic model

## Abstract

Combinatorial fusion algorithm (CFA) is a machine learning and artificial intelligence (ML/AI) framework for combining multiple scoring systems using the rank-score characteristic (RSC) function and cognitive diversity (CD). When measuring the relevance of a publication or document with respect to the 17 Sustainable Development Goals (SDGs) of the United Nations, a classification scheme is used. However, this classification process is a challenging task due to the overlapping goals and contextual differences of those diverse SDGs. In this paper, we use CFA to combine a topic model classifier (Model A) and a semantic link classifier (Model B) to improve the precision of the classification process. We characterize and analyze each of the individual models using the RSC function and CD between Models A and B. We evaluate the classification results from combining the models using a score combination and a rank combination, when compared to the results obtained from human experts. In summary, we demonstrate that the combination of Models A and B can improve classification precision only if these individual models perform well and are diverse.

## 1. Introduction

Powerful classification tools are used to help organize, search, and understand our increasingly digitized knowledge. Topic models have been used to categorize works in bioinformatics, for instance, among many other fields [1]. These tasks are challenging when there is a lack of sufficient well-labeled training data and when documents belong to multiple categories in different proportions [2]. This work shows how the classification precision of multi-category models with limited training data can be improved by combining different methodological approaches as a single classifier.

Combinatorial fusion algorithm (CFA) provides methods and algorithms for combining multiple scoring systems using the rank-score characteristic (RSC) function and cognitive diversity (CD) [3,4]. It has been used widely in protein structure prediction [5], ChIP-seq peak detection [6], virtual screening and drug discovery [7,8], target tracking [9], stress detection [10,11], portfolio management [12], visual cognition [13], wireless network handoff detection [14], combining classifiers with diversity and accuracy [15], and text categorization [16], to name just a few (see [17,18,19] and the references within).

This paper applies CFA to the novel challenge of measuring how the work of the United Nations system aligns with the 17 Sustainable Development Goals (SDGs) [20].

The SDGs are a set of concepts that are themselves interrelated, making classification challenging in the absence of a large training dataset. Creating such a dataset requires subjective decisions on the strength of the connection between any given term and each of the 17 goals. As a result, most attempts to map the connections between documents in the SDG space have been made by experts in the context of narrow research questions [21,22,23,24]. To deal with the scale and objectivity problem, two classification models have been proposed to classify documents according to the SDGs. Model A is a scoring system using the topic modeling method proposed by D. M. Blei [2,21]. Model B relies on the Semantic Web [25,26]. Both of these classifiers also overcome the lack of a training dataset in unique ways. Other proprietary tools that measure the alignment of activities, products, and services with the SDGs exist, but are not available for research use and are therefore not included in this analysis [27].

Having multiple classification systems available necessarily raises the question of how well they perform relative to some ground truth. Beyond this question, this paper is concerned with the additional performance that can be gained by combining diverse classification methodologies in such a way as to improve the performance beyond any individual method. The combinatorial fusion algorithm is shown to improve on the classification precision of both models by combining their results.

The CFA procedure combines data from different sources by converting them to a rank-score space. A scoring system A on the set of data items $D=\left\{d_{1},\;d_{2},\;\ldots ,\;d_{n}\right\}$, consists of a score function $s_{A}$ and a derived rank function $r_{A}$. By sorting the score values in the score function $s_{A}:\;D-->R$ in descending order and assigning a rank number to each of the n data items, the rank function $r_{A}:\;D-->N$ is obtained where N={1, 2, …, n}. The RSC function $f_{A}:\;N-->R$ is calculated as:

(1) $$f_{A}\left(i\right)=s_{A}\left(r_{A}{}^{-1}\left(i\right)\right)=\left(s_{A}\;\circ \;\;r_{A}{}^{-1}\right)\left(i\right),$$

by sorting the score values using the rank value as the key [3,4,18]. The notion of the RSC function was proposed by Hsu, Shapiro, and Taksa [4] in information retrieval. A similar notion was used in different contexts, such as urban development and computational linguistics [28,29].

Using this methodology, cognitive diversity, CD(A, B), between Models A and B is defined as:

(2) $$CD\left(A,\;B\right)=\sqrt{\sum \;{\left(f_{A}\left(i\right)-f_{B}\left(i\right)\right)}^{2}}$$

In this paper, we combine the SDG classification Models A and B using average score combination (SC(A, B)=S) and the sum of squared ranks combination (RC(A, B)=R), with:

(3) $$s_{S}\left(d_{i}\right)=\left(s_{A}\left(d_{i}\right)+s_{B}\left(d_{i}\right)\right)/2,$$

and,

(4) $$s_{R}\left(d_{i}\right)={\left(r_{A}\left(d_{i}\right)\right)}^{2}+{\left(r_{B}\left(d_{i}\right)\right)}^{2}$$

**Remark** **1.** *In cases when there are ties in the score or rank combination, that is, when*$s_{A}$*and*$s_{B}$*, or when*$r_{A}$*and*$r_{B}$*are exact opposites, we add a small random tie-breaker term c to the result of one of the two models, e.g., Model A,*$s_{R}\left(d_{i}\right)={\left(r_{A}\left(d_{i}\right)\right)}^{2+c}+{\left(r_{B}\left(d_{i}\right)\right)}^{2}$*, where −0.000001 > c > 0.000001*.

By sorting the score values of these two score functions, $s_{S}$ and $s_{R}$, into decreasing and increasing orders, respectively, the two rank functions for *S*, $r_{S}$, and for *R*, $r_{R}$, are obtained.

We evaluate the performance of each model using precision @*k*, *k* = 1, 3, 5, and 8. For each document (or publication in general), a human expert gives a scoring system H. A subset of *k* elements from *D*, denoted as Re(H) consisting of those SDGs which are ranked at top *k*, is considered as a relevant set of SDGs for the document. For Model A, the precision of *A* at rank *k* (pre@k) is the number of elements in the intersection of Re(A) and Re(H) divided by *k*.

In Section 2, we describe Models A and B in more detail. We characterize each of the models using the RSC function and then compute the cognitive diversity *CD*(*A*, *B*) for each document. Three examples are used to illustrate different values of cognitive diversity at low, middle, and high levels, *CD*(*A*, *B*) = 0.08, 0.46, and 1.67, respectively. We next evaluate the performance of score combination *SC*(*A*, *B*) and rank combination *RC*(*A*, *B*) models compared to the human-derived classification for nine test publications. In Section 3 we extend this analysis to an additional 30 publications chosen at random from the corpus of documents, and we show that combined models have performance advantages over individual classification models. Section 4 concludes the paper with a discussion of the results and implications.

## 2. Materials and Methods

The two models used to classify documents according to the SDGs use very different methodologies. Model A uses a machine learning clustering algorithm applied to a carefully selected representative sample of documents to generate a classifier [21]. Model B uses an ontology of terms and the semantic connections between those terms and the SDGs [25]. Each model is formally described below.

### 2.1. Model A

Model A uses a Latent Dirichlet allocation (LDA) algorithm to develop a probabilistic model of the 17 SDGs that can be used for classification [21]. LDA algorithms create semantically meaningful groupings from a collection of documents by considering documents as the result of a probabilistic sampling over the topics that describe the corpus, and over the words that comprise each topic [2].

Formally, the generative process for LDA is defined by the statistical assumptions of the dependencies in the joint distribution of the hidden and observed variables [2]:

(5) $$p\left(\beta_{1:K},\theta_{1:D},z_{1:D},w_{1:D}\right)=\overset{K}{\underset{i=1}{\prod }}p\left(\beta_{1}\right)\overset{D}{\underset{d=1}{\prod }}p(\theta_{d})\left(\overset{N}{\underset{n=1}{\prod }}p\left(z_{d,n}{|\theta }_{d}\right)p\left(w_{d,n}{|\beta }_{1:K},z_{d,n}\right)\right)$$

where $z_{d,n}$ is the topic assignment for word *n* in document *d*, and it depends on the topic proportions for each document, $\theta_{d}$. $w_{d,n}$ depends on the topic assignment and on all the topics $\beta_{1:K}$. Each $\beta_{K}$ is a distribution over the vocabulary. The computed topic structure, given a set of observed documents, $w_{1:D},$ is the above joint distribution divided by the marginal probability of seeing the observed corpus under any topic model, $p\left(w_{1:D}\right)$.

Training a classifier using LDA thus involves finding the best distribution of topics and words that would statistically recreate the training data. Once trained, each topic then comprises a list of words with individual probability weights that reflect the likelihood of being selected in a random draw. Words also belong to multiple topics with different probabilities.

By relying on the probabilistic nature of how the LDA algorithm assigns categories, it is possible to get around the need for an extensive labelled training dataset, one of the main requirements to train a traditional multi-class classifier. Creating labelled training data for SDG classification is difficult and costly since each of the 17 SDGs are combinations of multiple concepts and themes, as discussed in [21]. For instance, SDG 1 is broadly concerned with poverty, but includes targets on social protection, access to services, inequality, resilience, development cooperation, and policy frameworks.

It was shown that by selecting a group of sufficiently unique texts that reflect each of the 17 SDGs it is possible to use LDA to estimate a topic model with 17 precise topics (i.e., topics with smaller weights) [21]. Carefully selected groups of documents representing each SDG, when categorized using LDA, will result in a probabilistic model capable of differentiating among the 17 groups. Armed with this model, classifying out of sample texts, and inferring their SDG scores is equivalent to solving the question: given the word probabilities in each topic, what are the sampling probabilities from each of the 17 topics that minimize the difference between the result and the target document? The resulting 17 probability weights are interpreted as the SDG scores for each document.

### 2.2. Model B

Model B uses a semantic link approach to classify the SDG content of a given text without relying on a training dataset. This model relies on the Semantic Web to measure the connection between the content of a publication and each SDG. A description of the Semantic Web is available in [26] and a full description of the method for connecting ontologies and the SDGs is described in [25]. In short, a predetermined ontology of SDG terms formalizes the basic schema of the SDG goal-target-indicator-series hierarchy. This ontology allows the creation of a set of Internationalized Resource Identifiers (IRIs) for the SDGs, targets, and indicators. The following example illustrates the process:

1. Identify a keyword in the text: “… beaches estuaries dune systems mangroves MARSHES lagoons swamps reefs, etc., are …”;
2. The UNBIS concept extracted from the keyword via its synonym: WETLANDS;
3. The path from the extracted concept to the subject tag associated with the SDG entity: WETLANDS -> SURFACE WATERS -> WATER;
4. The most relevant goal associated with the subject tag WATER: “06 Ensure availability and sustainable management of water and sanitation for all”.

The result of this structured approach is the ability to effectively determine the relatedness of different items to the SDGs, helping to link unstructured documents to SDG concepts. Model B uses this structure to compute the frequency of selected concepts and the number of paths linking those concepts to the SDG entities in the semantic structure. The results are interpreted as the SDG scores in the same way as is done in Model A.

### 2.3. Performance Evaluation of Models A and B

The dataset used in this analysis comprises 267 texts published by the United Nations between 1995 and 2019. These include major flagship publications, reports by task teams, reports of the Secretary General, research notes, reports published by ECOSOC, thematic policy briefs, a full collection of DESA’s working papers, and other texts [21].

To test the performance of each classification system it is necessary to overcome the lack of a “true” objective SDG classification for each publication. This is particularly difficult in large texts that address many of the interrelated SDGs. It is possible, however, to compare the results of the algorithmic classifiers against the subjective opinion of a subject-matter expert. An example of how to apply this approach to measure how well the classifier performs in a sample as well as a subjective analysis of the results is provided in [21].

For this analysis we apply a more systematic evaluation of the classification methodologies. Initially, nine documents were selected from the corpus based on their computed cognitive diversity (CD) scores (see Equation (2)). Three documents represent the lowest CD scores, three represent median CD scores, and three represent the highest CD scores between Models A and B. These nine documents are evaluated by a human subject-matter expert, who ranked the importance of each of the SDGs in the text [30]. Below we discuss the individual results of Models A and B for one document from each of the low, median, and high cognitive diversity groups.

#### 2.3.1. Example 1: Low Cognitive Diversity

**Figure 1:**
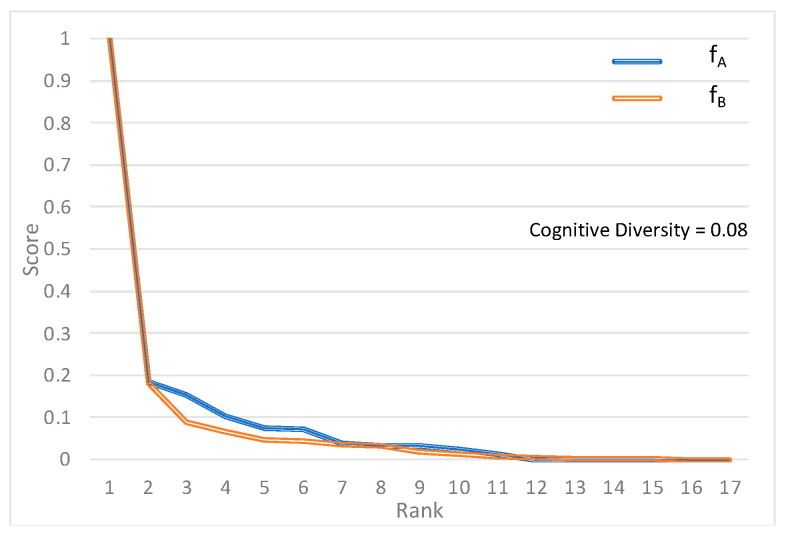
Rank-score functions *f_A_* and *f_B_* for low cognitive diversity case [30].

The document with the lowest cognitive diversity between A and B is titled “Behavioural Factors as Emerging Main Determinants of Child Mortality in Middle-Income Countries: A Case Study of Jordan”. Plotting the rank-score functions from using Model A and Model B illustrates the similarity of the scoring behavior of the two models (Figure 1). The classification models have nearly identical relationships between scores and ranks.

Table 1 shows the top eight SDGs according to each model, as well as according to a human subjective evaluation. The paper identifies the main determinants of child mortality in Jordan, so it is expected to be narrowly linked to SDG 3 (“good health and well-being”). Not surprisingly, both Model A and Model B reflect this and closely agree. Model A and Model B agree closely with the human-derived classification, as shown by the precision of the respective models for the top three, top five, and top eight categories.

#### 2.3.2. Example 2: Median Cognitive Diversity

**Figure 2:**
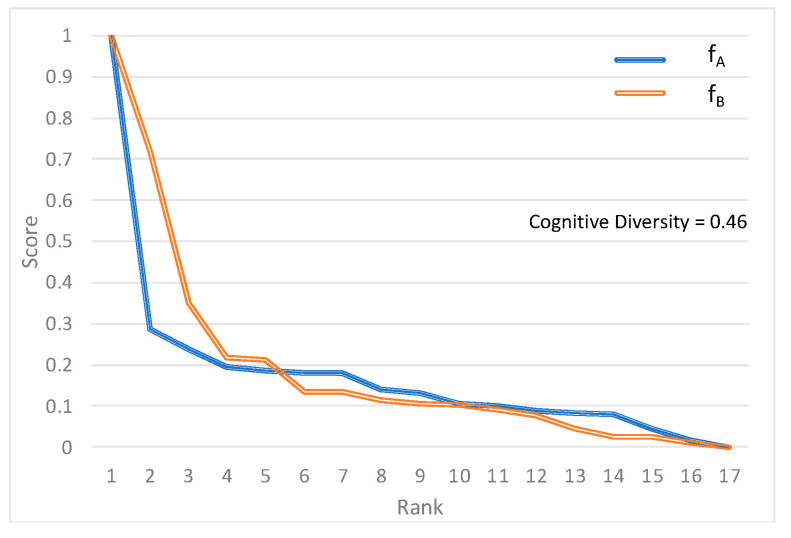
Rank-score functions *f_A_* and *f_B_* for median cognitive diversity case [30].

At the median of the distribution of the computed cognitive diversity scores, the rank-score functions for each model show a larger difference between Models A and B compared to the previous example (Figure 2). Nonetheless, there are no drastic differences in how the two classification models relate scores and ranks.

Table 2 shows the classifications derived from each of the models. This document is concerned with identifying links between the education goal (SDG 4) and various other goals. Both models correctly identify SDG 4 as the highest ranked classification but differ in the order of subsequent results. When compared with a human-classified ranking, the precision of Models A and B are different.

#### 2.3.3. Example 3: High Cognitive Diversity

**Figure 3:**
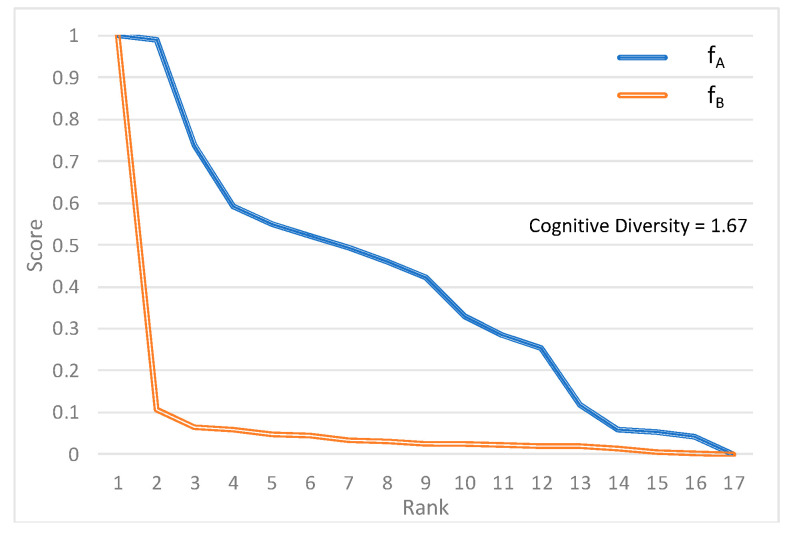
Rank-score functions *f_A_* and *f_B_* for high cognitive diversity case [30].

The largest difference between how each model ranks the classifications is for the thematic part of the 2002 World Economic and Social Survey, titled “Private-Public Interaction in Achieving Society’s Goals” (Figure 3). This is a book-length document with a complex thematic analysis about how public and private sectors produce the goods and services needed for development. With such a broad range of topics discussed, methodological differences between Models A and B are accentuated. The report discusses infrastructure and sectoral investments in energy, education, healthcare, and food production and includes a significant discussion on partnerships to achieve these goals.

Compared to the human opinion, neither model does very well in identifying the top SDG and both classify just one of the top three SDGs (Table 3). Model A, for instance, gives a very balanced distribution of the scores, identifies the language used for partnerships and cooperation throughout the report (SDG 17), and correctly captures the multi-thematic nature of the text. Model B, on the other hand, gives much more weight to the health SDG but at the expense of the other SDGs.

### 2.4. Results of Combining Models A and B for Nine Sample Documents

Having examined the performance of the individual models, we next evaluate the performance of the combined models with a small sample that allows us to peer into classification results in detail. We examine the classification results of using both combined models SC(A, B), by score combination, and RC(A, B), by rank combination, compared to the human-derived classification.

For the document with the lowest CD (Table 4), there is strong agreement on the top SDGs between the two combination classification models, and both models perform similarly compared to the human classification, as shown by the computed precision. The differences stem from slight variations in the classification order, and a disagreement between SDGs 10 and 16 in the top eight results. Given the narrow focus of the document, these results show an expected similarity between the various models.

For the document with the median CD (Table 5), there is a still strong, albeit smaller agreement on the top eight SDGs between the two combination models. The models also show a slightly larger difference in how they perform compared to the human-determined classification. There are more differences in the classification order of the first three and the first eight SDGs.

For the document with the highest CD (Table 6), the two models also perform similarly, though there is a significant difference in the choice of the first SDG. The top eight SDGs are the same in both combination models, but not their order.

### 2.5. Comparing the Performance of the Four Models

Table 7 compares the performance of all four models in classifying the three example documents (low, median, and high CD). We see that even though each of the individual models (A and B) does not perform steadily across the spectrum of documents (or publications), the combined models, SC(A, B) and RC(A, B), do perform consistently as well as, or better than, each of the two individual models. The classification precision of one of the combined models, when compared to the classification results obtained by human experts, is as high as or higher than what is achieved by the individual models.

Table 8 and Table 9 show the comparative performance of each of the combined models—SC(A, B) and RC(A, B)—relative to the most precise individual model (A or B). The results show that in most of the 36 calculated precisions, the combined model either matched or improved on the results of the individual models.

**Figure 4:**
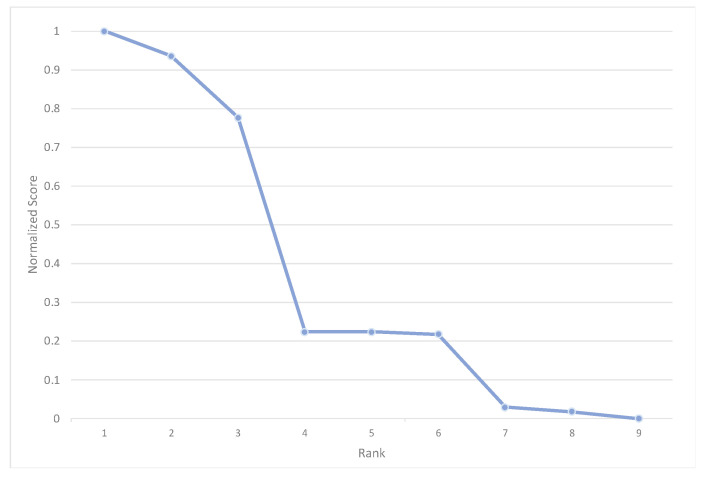
Rank-score functions of the cognitive diversity of the 9 test cases [30].

The rank-score function of the normalized cognitive diversity scores is also used to describe the distribution of CD scores in the corpus being analyzed, helping to identify breaks or formulate rules on how to select the best combined model to use (Figure 4). Using the nine documents as an example, the three highest normalized CD scores show a clear break from the other six cases. In addition, the six cases are clearly divided into two groups. Therefore, SC(A, B) is used for the lowest three CD cases, and RC(A, B) is used for the highest three CD cases. For the three cases with median CDs, either SC(A, B) or RC(A, B) can be used.

The collection of data from the domain-experts will be carried out through an email survey of staff in research institutions that focus on the Sustainable Development Goals, including the United Nations Department of Economic and Social Affairs. This collection will also create a singular testing dataset for use in evaluating and testing SDG classification models that will improve the accuracy of combinatorial fusion methodologies.

Table 10 shows how using such a decision rule influences the precision results. The table shows the difference in precision between the combined models and the individual models for each of the 36 computed precisions (9 documents × 4 precision levels). The gain in precision is measured in proportion to the number of classifications for a given precision level. For example, an improvement of a single classification under pre@3 results in a 1/3 gain, or 33%. The overall improvement is summarized at the end of the table also as a proportion of the total number of classifications. The results show how, except for the precision of the top classification, using this decision rule results in higher precision [30].

## 3. Extension of the Results to a Larger Set of Sample Documents

### 3.1. Extending by an Additional 30 Randomly Selected Sample Documents

**Figure 5:**
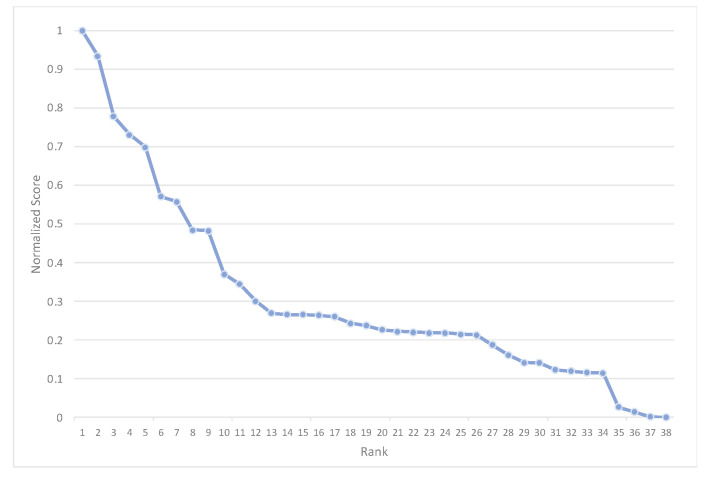
Rank-score functions of the cognitive diversity of the 38 test cases.

With the methods established in Section 2 we extend the evaluation of the performance of the score and rank combination models to an additional 30 documents, randomly selected from the full corpus of 267 texts. Together with the documents used in the preliminary analysis, the full evaluation sample includes 38 documents (one document from the limited sample was also selected in the random drawing). The rank-score function of the normalized cognitive diversity scores of the 38 documents shows a concave shape, indicating that the differences in how Models A and B score for a given rank are small except in a few cases. Only a third of the selected documents show a significant dispersion in their cognitive diversity scores, ranging from 1 to 0.3. The last two-thirds of the documents have CD scores below 0.3 (Figure 5).

We examine the performance of the score and rank combination models in relation to the human-determined classification and compare this performance with the best performing individual model (A or B). The results are shown with respect to the average precision of Models A and B and their cognitive diversity scores in the discussion below. The performance is analyzed according to each of the precision levels (1, 3, 5, and 8).

### 3.2. Combination Results in Terms of Precision @ 1

**Figure 6:**
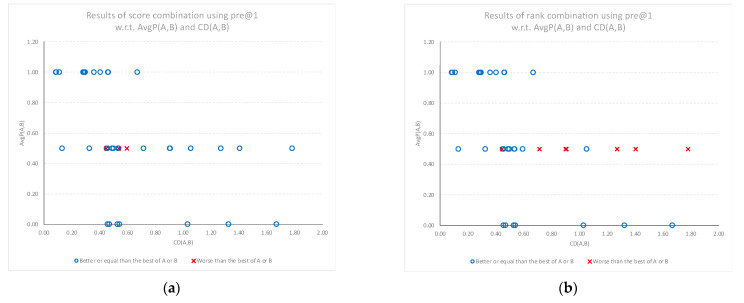
Relative performance of the combined models compared to the best of the individual models (best of A or B) for the top classification, plotted against the average performance (AvgP) of the individual models and cognitive diversity. (**a**) Results when using score combination *SC*(*A*, *B*). (**b**) Results when using rank combination *RC*(*A*, *B*).

Figure 6a,b shows the precision gains for the top classification (pre@1) using score combination *SC*(*A*, *B*) and rank combination *RC*(*A*, *B*), respectively. The overwhelming majority of the combination results show an equal or better performance (shown as a “o”) than the best individual model. These results hold regardless of the average performance of the individual models (*y*-axis). At this level of precision, rank combination results in a lower performance (shown as an “x”) for a wide range of cognitive diversity. The performance of the models determining the top classification is very sensitive to any misses (all or nothing), and the distribution of gains shows a mix of positive, neutral, and negative results for both combination models.

### 3.3. Combination Results in Terms of Precision @ 3

**Figure 7:**
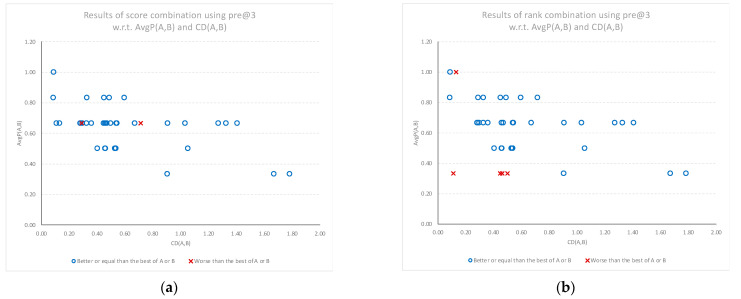
Relative performance of the combined models compared to the best of the individual models (best of A or B) for the top three classifications, plotted against the average performance (AvgP) of the individual models and cognitive diversity. (**a**) Results when using score combination *SC*(*A*, *B*). (**b**) Results when using rank combination *RC*(*A*, *B*).

The performance gains from using the score and rank combination models compared to the best performing individual model are more evident when examining the precision of the top three classifications. Figure 7a,b shows that for documents with higher cognitive diversity scores, the performance gains of using combined models are consistently more positive. The rank combination results also show that the cases with worse performance results are clustered where the individual models have a low average performance, indicating some consistent divergence between both model outcomes and human classification.

### 3.4. Combination Results in Terms of Precision @ 5

**Figure 8:**
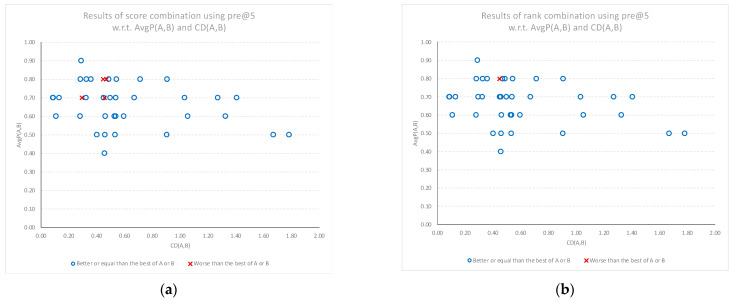
Relative performance of the combined models compared to the best of the individual models (best of A or B) for the top five classifications, plotted against the average performance (AvgP) of the individual models and cognitive diversity. (**a**) Results when using score combination *SC*(*A*, *B*). (**b**) Results when using rank combination *RC*(*A*, *B*).

Figure 8a,b shows the performance gains of *SC*(*A*, *B*) and *RC*(*A*, *B*) models for the top five classifications, in comparison to the average performance of each individual model. The results where the combination models underperformed the best of the individual models are plotted as a red “x”, while the results that matched or outperformed the best individual model are plotted as a blue circle. Again, the results show that positive gains are more prevalent than negative gains, and that any regression in performance happens at lower cognitive diversity scores. There is also some indication that using a rank combination at this level of precision results in better overall results with fewer cases of lower performance.

### 3.5. Combination Results in Terms of Precision @ 8

**Figure 9:**
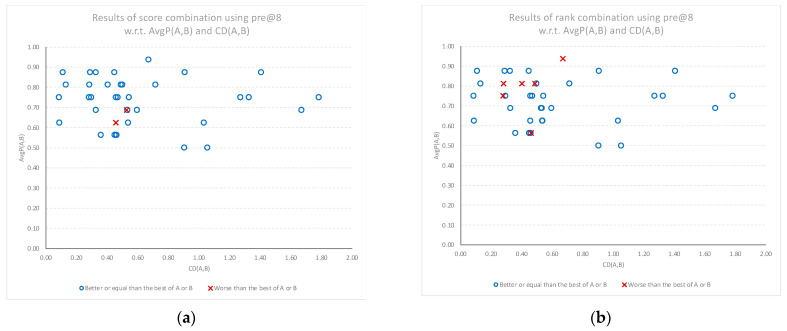
Relative performance of the combined models compared to the best of the individual models (best of A or B) for the top eight classifications, plotted against the average performance (AvgP) of the individual models and cognitive diversity. *N* = 38 documents. (**a**) Results when using score combination *SC*(*A*, *B*). (**b**) Results when using rank combination *RC*(*A*, *B*).

Finally, when considering the top eight classifications, the performance gains from using the score and rank combination models, compared to the best performing individual model, is again confirmed. Figure 9a,b once again shows the performance gains of the combination models *SC*(*A*, *B*) and *RC*(*A*, *B*), respectively, in comparison to the average performance of each individual model. The results show a consistent positive gain in performance for all levels of average individual model performance. As in the previous results, the gains are stronger as cognitive diversity increases, with the only negative performance results at smaller cognitive diversity levels.

## 4. Discussion

In this paper, we used combinatorial fusion algorithm (CFA), including the rank-score characteristic (RSC) function and cognitive diversity (CD), to combine two models (A and B) to improve the performance of the classification scheme. In particular, the RSC function of a model on a document can depict the ranking (or scoring) behavior (or pattern) of the model and help identify any systemic behavior that is the result of the methodological approach used. In addition to that, cognitive diversity *CD*(*A*, *B*) is used to measure the difference between A and B. The distribution of *CD*(*A*, *B*) for the 38 publications serves as a guiding principle to use either rank combination or score combination (Figure 4 and Figure 5).

The two analytical measures, RSC function and CD, that emerge from applying the CFA, are helpful to the researcher who is investigating the impact of methodological choice on classification results within a given domain. The score-combination or rank-combination fusion models made possible by the CFA are shown to match or outperform the individual models in subjective tests that compare them to the opinion of a domain expert. The results of the current paper are in line with those findings. Namely, the pattern matches the results from three other recent publications [1,15,31] where model fusion and cognitive diversity were used to perform combinatorial fusion. The combined models perform consistently as well as, and in many cases outperform, the best of the two individual models. Other relevant publications include those discussing the rank space [18,32] and those in the field of metric fixed point theory that can be useful to further this methodology [33,34].

## 5. Conclusions

In summary, we demonstrate that a combination of the two models can improve each individual model only if these two models are relatively good (in terms of performance ratio) and they are diverse (in terms of cognitive diversity). In addition to that, model fusion using combinatorial fusion algorithms was able to improve not only the prediction but also the data quality with regard to reproducibility by subject experts.

Future work includes the following: (a) derive combined models using a weighted combination by the performance or by the diversity strength of each model which is a different and useful measure of the diversity between the attributes and algorithms, (b) expand this analysis to more than two individual models by including the results of the newly released “SDG Meter” classification model created by the United Nations Environment Programme (UNEP), and (c) use multi-layer combinatorial fusion (MCF) to derive a sequence of combined models on a rank space. Further work also includes the formalization of a decision rule and of validation tests by creating a larger testing dataset of domain-expert document classifications. Moreover, we will investigate the sensitivity of both rank and score combinations to precision @ 1 with respect to any specific publications. The collection of data from the domain-experts will be carried out through an email survey of staff in research institutions that focus on the Sustainable Development Goals, including the United Nations Department of Economic and Social Affairs. This collection will also create a singular testing dataset for use in evaluating and testing SDG classification models that will improve the accuracy of combinatorial fusion algorithms.

## Figures and Tables

**Table 1:** Ranking results of Model A and Model B as well as human evaluation for low cognitive diversity case.

Rank	Results		
	SDG of Model A	SDG of Model B	Human
1	d_3	d_3	d_3
2	d_4	d_5	d_4
3	d_5	d_2	d_5
4	d_17	d_4	d_1
5	d_6	d_16	d_2
6	d_10	d_6	d_6
7	d_2	d_11	d_10
8	d_13	d_17	d_16
precision @ 1	1.00	1.00	
precision @ 3	1.00	0.67	
precision @ 5	0.60	0.80	
precision @ 8	0.75	0.75	

Source: [30].

**Table 2:** Ranking results of Model A and Model B as well as human evaluation for median cognitive diversity case.

Rank	Results		
	SDG of Model A	SDG of Model B	Human
1	d_4	d_4	d_4
2	d_12	d_3	d_8
3	d_13	d_5	d_5
4	d_15	d_8	d_12
5	d_17	d_16	d_10
6	d_5	d_6	d_3
7	d_8	d_17	d_1
8	d_11	d_11	d_13
precision @ 1	1.00	1.00	
precision @ 3	0.33	0.67	
precision @ 5	0.40	0.60	
precision @ 8	0.63	0.50	

Source: [30].

**Table 3:** Ranking results of Model A and Model B as well as human evaluation for high cognitive diversity case.

Rank	Results		
	SDG of Model A	SDG of Model B	Human
1	d_17	d_3	d_8
2	d_4	d_2	d_7
3	d_3	d_4	d_3
4	d_12	d_11	d_4
5	d_9	d_5	d_2
6	d_16	d_17	d_9
7	d_7	d_6	d_17
8	d_10	d_8	d_12
precision @ 1	0.00	0.00	
precision @ 3	0.33	0.33	
precision @ 5	0.40	0.60	
precision @ 8	0.75	0.63	

Source: [30].

**Table 4:** Ranking results of Models *SC*(*A*, *B*) and *RC*(*A*, *B*) as well as human evaluation for low cognitive diversity case: *CD*(*A*, *B*) = 0.08.

Rank	Results		
	SDG of *SC*(*A*, *B*)	SDG of *RC*(*A*, *B*)	Human
1	d_3	d_3	d_3
2	d_5	d_5	d_4
3	d_4	d_4	d_5
4	d_17	d_2	d_1
5	d_2	d_6	d_2
6	d_6	d_17	d_6
7	d_10	d_11	d_10
8	d_11	d_16	d_16
precision @ 1	1.00	1.00	
precision @ 3	1.00	1.00	
precision @ 5	0.80	0.80	
precision @ 8	0.75	0.75	

Source: [30].

**Table 5:** Ranking results of Models *SC*(*A*, *B*) and *RC*(*A*, *B*) as well as human evaluation for median cognitive diversity case: *CD*(*A*, *B*) = 0.46.

Rank	Results		
	SDG of *SC*(*A*, *B*)	SDG of *RC*(*A*, *B*)	Human
1	d_4	d_4	d_4
2	d_3	d_5	d_8
3	d_5	d_8	d_5
4	d_12	d_17	d_12
5	d_8	d_12	d_10
6	d_17	d_11	d_3
7	d_13	d_6	d_1
8	d_11	d_13	d_13
precision @ 1	1.00	1.00	
precision @ 3	0.67	1.00	
precision @ 5	0.80	0.80	
precision @ 8	0.75	0.63	

Source: [30].

**Table 6:** Ranking results of Models *SC*(*A*, *B*) and *RC*(*A*, *B*) as well as human evaluation for high cognitive diversity case: *CD*(*A*, *B*) = 1.67.

Rank	Results		
	SDG of *SC*(*A*, *B*)	SDG of *RC*(*A*, *B*)	Human
1	d_13	d_1	d_13
2	d_1	d_9	d_12
3	d_9	d_12	d_9
4	d_12	d_7	d_11
5	d_7	d_13	d_10
6	d_11	d_14	d_1
7	d_8	d_8	d_8
8	d_14	d_11	d_7
precision @ 1	1.00	0.00	
precision @ 3	0.67	0.67	
precision @ 5	0.60	0.60	
precision @ 8	0.88	0.88	

Source: [30].

**Table 7:** Precision results of Models A, B, *SC*(*A*, *B*) and *RC*(*A*, *B*) for low, median, and high cognitive diversity.

	Pre@k (*A*)	Pre@k (*B*)	Pre@k *SC*(*A*, *B*)	Pre@k *RC*(*A*, *B*)
**Low CD case**				
*k* = 1	1.00	1.00	1.00	1.00
*k* = 3	1.00	0.67	1.00	1.00
*k* = 5	0.60	0.80	0.80	0.80
*k* = 8	0.75	0.75	0.75	0.75
**Median CD case**				
*k* = 1	1.00	1.00	1.00	1.00
*k* = 3	0.33	0.67	0.67	1.00
*k* = 5	0.40	0.60	0.80	0.80
*k* = 8	0.63	0.50	0.75	0.63
**High CD case**				
*k* = 1	0.00	0.00	0.00	0.00
*k* = 3	0.33	0.33	0.33	0.33
*k* = 5	0.40	0.60	0.60	0.60
*k* = 8	0.75	0.63	0.88	0.88

Source: [30].

**Table 8:** Difference between precision results of Model *SC*(*A*, *B*) and the best of Models A or B for all test cases.

Cognitive Diversity	Gain in Pre @ 1	Gain in Pre @ 3	Gain in Pre @ 5	Gain in Pre @ 8
**0.08**	0.00	0.00	0.00	0.00
**0.11**	0.00	0.00	0.20	0.00
**0.13**	0.00	0.33	0.00	0.00
**0.45**	−1.00	0.00	−0.20	0.00
**0.46**	0.00	0.00	0.20	0.125
**0.46**	0.00	0.00	0.40	0.00
**1.40**	−1.00	0.33	0.00	0.00
**1.67**	0.00	0.00	0.00	0.125
**1.78**	0.00	0.33	0.00	0.125
**Avg Improvement**	**−22%**	**11%**	**6.7%**	**4.2%**

Source: [30].

**Table 9:** Difference between precision results of Model *RC*(*A*, *B*) and the best of Models A or B for all test cases.

Cognitive Diversity	Gain in Pre @ 1	Gain in Pre @ 3	Gain in Pre @ 5	Gain in Pre @ 8
**0.08**	0.00	0.00	0.00	0.00
**0.11**	0.00	−0.33	0.00	0.00
**0.13**	0.00	0.33	0.00	0.00
**0.45**	−1.00	−0.33	0.00	0.125
**0.46**	0.00	0.33	0.20	0.00
**0.46**	0.00	−0.67	0.20	0.00
**1.40**	−1.00	0.33	0.00	0.00
**1.67**	0.00	0.00	0.00	0.125
**1.78**	−1.00	0.33	0.00	0.125
**Avg Improvement**	**−33%**	**0%**	**4.4%**	**4.2%**

Source: [30].

**Table 10:** Difference between precision results of combined and individual models using *RC*(*A*, *B*) for the highest three cognitive diversity tests, and *SC*(*A*, *B*) for the lowest six cognitive diversity tests.

Cognitive Diversity	Gain in Pre @ 1	Gain in Pre @ 3	Gain in Pre @ 5	Gain in Pre @ 8
**0.08**	0.00	0.00	0.00	0.00
**0.11**	0.00	0.00	0.20	0.00
**0.13**	0.00	0.33	0.00	0.00
**0.45**	−1.00	0.00	−0.20	0.00
**0.46**	0.00	0.00	0.20	0.125
**0.46**	0.00	0.00	0.40	0.00
**1.40**	−1.00	0.33	0.00	0.00
**1.67**	0.00	0.00	0.00	0.125
**1.78**	−1.00	0.33	0.00	0.125
**Avg Improvement**	**−33%**	**11%**	**6.7%**	**4.2%**

Source: [30].

## Data Availability

The data presented in this study are available on request from the corresponding author. The data are not publicly available due to the use of copyrighted or otherwise restricted access sources.
